# Underestimation of weight and its associated factors among overweight and obese adults in Pakistan: a cross sectional study

**DOI:** 10.1186/1471-2458-11-363

**Published:** 2011-05-23

**Authors:** Seema Bhanji, Ali Khan Khuwaja, Fawad Siddiqui, Iqbal Azam, Khawar Kazmi

**Affiliations:** 1 Department of Family Medicine, The Aga Khan University, Stadium Road, PO Box 3500, Karachi - 74800, Pakistan; 2 Department of Community Health Sciences, The Aga Khan University, Stadium Road, PO Box 3500, Karachi - 74800, Pakistan; 3 Section of Cardiology, Department of Medicine, The Aga Khan University, Stadium Road, PO Box, 3500, Karachi - 74800, Pakistan

## Abstract

**Background:**

Weight loss is known to decrease the health risks associated with being overweight and obese. Awareness of overweight status is an important determinant of weight loss attempts and may have more of an impact on one's decision to lose weight than objective weight status. We therefore investigated the perception of weight among adults attending primary care clinics in Karachi, Pakistan, and compared it to their weight categories based on BMI (Body Mass Index), focusing on the underestimation of weight in overweight and obese individuals. We also explored the factors associated with underestimation of weight in these individuals.

**Methods:**

This was a cross sectional study conducted on 493 adults presenting to the three primary care clinics affiliated with a tertiary care hospital in Karachi, Pakistan. We conducted face to face interviews to gather data on a pre-coded questionnaire. The questionnaire included detail on demographics, presence of comorbid conditions, and questions regarding weight assessment. We measured height and weight of the participants and calculated the BMI. The BMI was categorized into normal weight, overweight and obese based on the revised definitions for Asian populations. Perception about weight was determined by asking the study participants the following question: Do you consider yourself to be *a*) thin *b*) just right *c*) overweight *d*) obese. We compared the responses with the categorized BMI. To identify factors associated with underestimation of weight, we used simple and multiple logistic regression to calculate crude odds Ratios (OR) and adjusted Odds Ratios (AOR) with 95% Confidence Intervals.

**Results:**

Overall 45.8% (n = 226) of the study participants were obese and 18% (n = 89) were overweight. There was poor agreement between self perception and actual BMI (Kappa = 0.24, SE = 0.027, p < 0.001). Among obese participants a large proportion (73%) did not perceive themselves as obese, although half (n = 102) of them thought they may be overweight. Among the overweight participants, half (n = 41) of them didn't recognize themselves as overweight. Factors associated with misperception of weight in overweight and obese participants were age ≥ 40 years (AOR = 3.4; 95% CI: 1.8-6.4), male gender (AOR = 2.97; 95% CI: 1.6-5.5), being happy with ones' weight (AOR = 6.4; 95% CI: 3.4-12.1), and not knowing one's ideal weight (AOR = 2.45, 95% CI: 1.10-5.47).

**Conclusion:**

In this cross sectional survey, we observed marked discordance between the actual and perceived weight. Underestimation of individual weight was more common in older participants (≥ 40 years), men, participants happy with their weight and participants not aware of their ideal weight. Accurate perception of one's actual weight is critical for individuals to be receptive to public health messages about weight maintenance or weight loss goals. Therefore educating people about their correct weight, healthy weights and prevention of weight gain are important steps towards addressing the issue of obesity in Pakistan.

## Background

The global epidemic of overweight and obesity - termed "globesity" is a major public health problem in the developed as well as the developing world. Rates of obesity have tripled in developing countries over the last 20 years, where the prevalence of overweight adults is in the range of 10 to 25%, and the prevalence of obesity ranges from 2 to 10% [1]. According to World Health Organization (WHO) estimates, 25.5 % of women and 18.8% of men in Pakistan are overweight (Body Mass index (BMI)

≥ 25) and 3.6% of women and 1 % of men are obese (BMI ≥ 30) [2]. Additionally, the prevalence of overweight/obese individuals is estimated to be much higher (56% in men and 67% in women) in urban settings if the revised definitions for Asian populations are used to categorize weight [3]. The higher prevalence reported is probably because of lower thresholds used to categorize overweight and obese individuals in this population and also because of the rapidly developing epidemic of obesity in Pakistan.

Being obese or overweight increases the risk of chronic diseases like cardiovascular diseases, type 2 diabetes mellitus, a variety of cancers and death and it is closely related to the BMI status [4]. Weight loss has shown to decrease these health risks associated with being overweight and obese. Even a modest amount of weight loss has beneficial effects on hypertension and diabetes [5]. Awareness of weight status is an important determinant in weight loss attempts. Behavior change theories suggest that advice given to overweight people may go unheeded if they do not consider themselves to be overweight or obese. The trans-theoretical model of behavior change suggests that people are able to progress from pre-contemplation to contemplation when they are aware of their weight status coupled with the knowledge of benefit derived from weight loss [6]. The health belief model also suggests that perception of weight along with susceptibility to disease helps in behavior change intervention [6]. Many population based studies have observed that overweight and obese individuals tend to underestimate their weight status [7-9]. Research studies have also shown that awareness of being overweight/obese is an essential factor for weight loss attempts which may have more impact than objective weight status [10].

No studies have been conducted to date on weight perception in Pakistan. To reduce the growing number of obese/overweight people in the population, and to help them lose weight, it is important to first understand if there are problems with weight perception in Pakistan as have been observed globally, and what factors affect this awareness of weight perception. We investigated the perception of weight among adults attending primary care clinics and compared it to their weight categories based on BMI, particularly the underestimation of weight in overweight/obese individuals. We also explored the factors associated with underestimation of weight in these individuals.

## Methods

### Setting and sampling

We conducted a cross sectional study at three primary care clinics affiliated with a tertiary care hospital in Karachi, Pakistan. These clinics were selected for their location in three different geographical areas of Karachi, to include people with diverse socioeconomic and ethnic backgrounds. A non-probability convenience sample was drawn from adult (aged 18 year and above) patients or attendants visiting the primary health care units between March 2009 to July 2009. We explained the objective of the study to the study participants and obtained their informed consent. We also obtained permission for data collection at these sites.

The study protocol was developed in accordance with the Helsinki declaration. The study protocol and questionnaire were reviewed and approved by the Research Committee, Department of Family Medicine, Aga Khan University, Karachi. We provided the study participants with a consent form in Urdu, detailing the aims of the study, methods, institutional affiliations of the researchers, the anticipated benefits, the right to refuse, voluntary participation and the right to withdraw without any effect on the clinical care. Investigators obtained verbal consent which was documented on a separate sheet along with the daily log of the patient (approached, consented and interviewed).

The questionnaires were anonymous. The questionnaires were entered into a database by a data collecter, a trained a medical student. The data collecter ensured that the interviews and information collected were confidential. Access to the final data set was restricted to the principal investigator.

### Instrument

We designed a structured questionnaire in English after extensive literature search. The question on weight perception was modified from the questions used in previous studies by Johnson-Taylor, Gutierrez-Fisac and Howard [8,11,12]. The questionnaire was translated into Urdu and translated back into English. It was then piloted on small number of patients attending these clinics.

### Procedure

All adults presenting to the clinic were approached by the data collecter. The objective of the study was explained and consent was sought for participation. Once consent was obtained, a face to face interview was conducted by the data collecter using the structured questionnaire. The weight (in kilograms) and height (in centimeters) of the study participants was measured by the nursing staff of the clinic in a confidential manner.

BMI was calculated from weight and height measurements and was categorized into Normal weight: BMI = 18.5-22.9, Overweight: BMI = 23-24.9 and Obese BMI ≥ 25.0 based on the Asian thresholds for BMI categorization [13]

### Dependent variable

Perception regarding weight was determined by asking the following question: Do you consider yourself as *a*) thin *b*) just right *c*) overweight *d*) obese. The responses were then compared to the calculated BMI. For overweight participants, underestimation of overweight status was considered when the response was *a*) thin or *b*) just right. For obese participants, underestimation for obesity was considered when the response to the same question was *a*) thin or *b*) just right or *c*) overweight. Participants were interviewed prior to their physical measurements to reduce the bias in reporting misperception

### Independent variables

#### Participant demographics

This consisted of age, sex, highest level of education received and location of clinic visited.

#### Presence of co- morbid conditions

Participants were asked if they had diabetes, hypertension, ischemic heart disease or dyslipidemia.

#### Weight related assessment

Participants were asked if they had checked their weight in the last two years, if they had been advised about weight by any health professionals and if they knew their ideal body weight. Participants were asked to describe how they felt about their weight using the following descriptors; *a*) happy, *b*) unhappy or *c*) don't think about it.

### Statistical analysis

We calculated descriptive statistics (mean and standard deviations) to understand the basic characteristics of the study participants. Self perception of weight status was compared to the calculated BMI to determine the proportion of population exhibiting misperception. Kappa was calculated by comparing self perception to calculated BMI.

For model building, using underestimation of weight as a dependent variable, univariate analysis using simple logistic regression was carried out to look for any association with independent variables (age, sex, education, presence of co-morbid conditions, frequency of weight check, knowledge of ideal weight, weight advice received from health professional and feelings about weight). The results are reported as crude odds ratios (OR) with 95% confidence intervals (CI). Independent variables were then checked for multicollinearity; none of the variables were found to be correlated. Final model building was done by adding all variables in order of significance using multiple logistic regression and results are reported as adjusted Odds Ratios (AOR) with 95% CI. SPSS v 16.0 was used for data analysis.

## Results

We approached 560 people, out of which 60 did not consent (response rate 89%). Seven questionnaires were incomplete for anthropometric measurements, therefore 493 questionnaires were analyzed. Almost half (45.8%; 95%CI: 41.4-50.2) of the participants were obese and one fifth (18%; 95% CI: 14.7-21.5) were overweight.

The distribution of sociodemographic and personal characteristics of the study population are shown in Table 1. There were no statistical differences found according to the confidence intervals with regards to location of clinics, gender, level of education, knowledge of one's ideal weight, weight checked in last two years and presence of comorbid conditions for overweight/obese people. Rest of the characteristics were found statistically significant for overweight/obese people using the same criteria, which were higher for aged ≥40 years, being unhappy about weight and weight advice given by health professionals.

**Table 1:** Sociodemographic and personal characteristics of the study population

Characteristics	All n = 493	% Overweight/obese (95% CI) n = 315
**Location**		
Malir/Korangi	144	64.6 (54.9-74.3)
Clifton	200	69.0 (61.3-76.7)
Community Health Centre	149	56.4 (45.8-67.0)

**Age**		
< 40	309	54.7 (47.2-62.2)
≥ 40	184	79.3 (72.7-85.9)

**Gender**		
Male	317	66.6 (60.2-73.0)
Female	176	59.1 (49.7-68.5)

**Educational status (n = 489)**		
Illiterate	23	78.3 (59.3-97.3)
Undergrad	160	59.4 (49.5-69.3)
Graduate	244	65. 6 (58.2-73.0)
Postgraduate	62	61.3 (45.8-76.8)

**Feelings about weight**		
Happy/Don't think	337	57 (50-64)
Unhappy	156	78.8 (71.6-86.0)

**Knowledge of ones's ideal weight**		
Don't know weight	399	65.9 (60.2-71.6)
Know weight	94	55.3 (41.8-68.8)

**Weight checked in last two years**		
Not checked	73	61.6( 47.4-75.8)
Checked	420	64.3 (58.6-70.01)

**Weight advice given by health professionals**		
Not given	354	57.1 (50.3-63.9)
Given	139	81.3 (74.1-88.5)

**Presence of comorbid conditions** **(DM, HTN, dyslipidemia)**		
Yes	124	58.1 (46.7-69.5)
No	369	65.9 (59.9-71.9)

**Figure 1:**
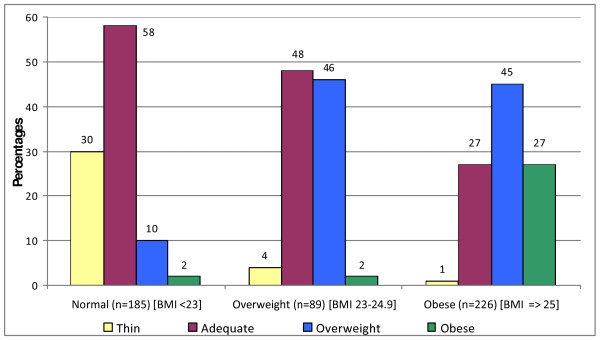
**Self perception of weight compared with the calculated BMI**.

Self perception of weight was compared to the calculated BMI (shown in Figure 1). Among obese participants 165 (73%) individuals did not perceive themselves as obese, although half (n = 102) of them thought they may be overweight. Among overweight participants, 41 (46%) of them didn't identify themselves as being overweight. Poor agreement was observed between self perception and actual BMI (Kappa = 0.24, SE = 0.027, p < 0.001). More males than females misperceived their weight both in the overweight (57% *vs *41%) and obese (84% *vs *52%) categories.

Factors associated with underestimation of weight in overweight and obese participants are presented in Table 2. In the univariate model, age, gender, being happy/not thinking about one's weight and comorbidity were associated with misperception. In the multivariate model age, gender, being happy/not thinking about one's weight and not knowing one's ideal weight were associated with misperception.

**Table 2:** Factors associated with underestimation of weight in overweight/ obese individuals

Variables	Misperceived n (%)	Crude Odds ratio (95% CI)	Adjusted Odds ratio (95% CI)
**Age**			
≥ 40	110 (75.3)	1.91 (1.17-3.11)**	3.40 (1.81-6.39)***
< 40	104 (61.5)	1	

**Gender**			
Male	162 (76.8)	3.31 (2.01-5.45) ***	2.97 (1.62 -5.44)***
Female	52 ( 50)		

**Educational status**			
Illiterate	12 (66.7)	0.81 (0.24-2.72)	0.53 (0.12-2.31)
Undergrad	60 (63.2)	0.70 (0.31-1.58)	0.53 (0.19-1.42)
Graduate	113 (70.6)	0.98 (0.45-2.13)	0.96 (0.37-2.43)
Postgraduate	27 (71.1)	1	

**Feelings about weight**			
Happy/Don't think	159 (82.8)	5.96 (3.55-9.99) ***	6.41 (3.38-12.16)***
Unhappy	55 (44.7)	1	

**Knowledge of ones's ideal weight**			
Don't know weight	39 (75)	1.51 (0.77-2.97)	2.45 (1.10-5.47)*
Know weight	175 (66.5)	1	

**Weight checked in last two years**			
Not checked	32 (71)	1.19 (0.60-2.38)	1.37 (0.59-3.16)
Checked	182 (67.4)	1	

**Weight advice given by health professionals**			
Not given	142 (70.3)	1.35 (0.83-2.2)	0.74 (0.39-1.42)
Given	72 (63.7)	1	

**Presence of comorbid conditions **(DM, HTN, dyslipidemia)			
Yes	59 (81.9)	2.58 (1.34-4.96)**	1.59 ( 0.75-3.39)
No	155 (63.8)	1	

*** p value < 0.001, ** p value < 0.01, * p value < 0.05

## Discussion

Misperception of weight status was highly prevalent in our study population. We observed that a substantial proportion of individuals in the overweight and obese categories inaccurately classified their weight status; almost half of the overweight and most of the obese participants misperceived their weight status. Appropriate perception of weight is strongly associated with weight loss efforts across genders and BMI categories [10]. Underestimation of weight status contributes to denial or minimization of current weight being a health risk and thus contributes to increase in health problems associated with obesity due to a failure to respond to health professional's advice [14-16].

Higher proportions of misperception have been reported previously. A study from an urban family medicine centre in United States reported similar proportion of misperception of obesity (BMI > 30) in participants coming for screening health checkups [17]. A nationally representative sample from Australia reported half of men and one quarter of women do not consider themselves to be overweight/obese [18]. A community sample from Australia also reported 66% of obese (BMI > 30) and 34% of overweight (BMI > 25) participants failing to recognize their correct weight status [12].

An important finding of our study is that a large number of overweight participants did not categorize themselves as overweight. Similar findings have been reported from a nationally representative sample of Spanish adults [11] and a community sample in Australia [12]. Being overweight (BMI = 23-24.9) is also risk factor for health related problems. This is also true in South Asian cultures, putting them at risk of excessive weight gain and associated health risks [19]. Being overweight is a precursor to obesity and it is relatively easier to lose weight at these moderate levels than at higher levels of weight gain. Thus overweight people should be educated about the associated health risks of being overweight and advised about appropriate weight loss strategies.

We found that being happy or not thinking about their weight was most strongly associated with weight misperception. A large proportion of overweight/obese participants in our study were either content or not concerned about their weight. Anderson et.al. found half of the overweight and obese women in their study to be satisfied with their body size in a national survey of overweight and obese women in United States [20] and Green et. al. observed a large proportion of overweight/obese men to be content with their weight in a nationally representative sample of Canadian adults [21]. This can be explained by social and cultural factors which influence the standards accepted for weight [22]. Traditionally body weight has been regarded as a symbol of health, prosperity and wealth in various populations [23]. This is also true in South Asian culture where people still consider weight to be associated with good health and wealth. They would therefore tend to accept overweight/obesity as a norm and are thus at higher risk of not perceiving it to be of any concern

Comparison between genders showed that a large proportion of men did not consider themselves to be overweight/obese compared with women. Similar trends have been observed in earlier population based studies [8,24]. This difference in perception again could be the result of social or cultural factors. Social and family pressures to maintain an acceptable body image affect women more than it affects men; consequently women are more sensitive of their weight status and they perceive their weight more accurately than men do. There is epidemiological evidence from Western countries that show an increasing trend of obesity in men [25], and the same trend may now be emerging in developing countries as well.

In our study, men and women aged 40 years and over were more likely to misperceive their weight status, which is in concordance with other studies [26,27]. Possible reasons may be that in Pakistan, older people are less concerned about their body image than younger people, which may alter their perception of weight. It may also be related to the increased prevalence of overweight and obesity in older age groups, which is usually accepted as an age related phenomenon [28]. This is a cause of concern because accumulation of risk factors with advancing age can increase the likelihood of chronic diseases.

Another important finding of our study associated with misperception is inadequate knowledge of ideal body weight which may lead the obese/overweight people to consider themselves of ideal weight. A study by Kuk et al observed higher ideal weight to be associated with greater body satisfaction and lower intention to participate in weight loss activities [29].

Presence of comorbid conditions lead to increased susceptibility and thus increased awareness of being overweight and obese, but an opposite phenomenon has been observed in our study. This may be because people having hypertension/dyslipidemia/diabetes mellitus may focus more on their primary illness and overlook their weight status. Also they may not consider obesity as a risk factor for chronic disease. Similar findings of poor perception of weight in people with diabetes and coronary heart disease have been observed in a community sample in Cracow, Poland [27]. Powell et al have reported about the weight misperception in people with comorbid conditions in a multi-ethnic urban cohort in Dallas, United states [30]. This weight misperception along with unhealthy life styles contributes to development and progression of chronic disease. This makes it imperative that the high risk groups correctly perceive their weight as knowledge of health risk associated with obesity alone may not prompt attempts to lose weight. Kruger et al., observed in a study of US adults that despite the knowledge of benefits associated with weight loss only half of the obese participants attempted to lose weight [31]. Awareness of one's weight did not improve weight perception among the participants in our study. Possible explanation may be the social or cultural factors that tolerate a higher body weight and inadequate knowledge of ideal body weight.

A large proportion of overweight/obese participants (64%, n = 202) in our study reported not being advised about their weight; only one third (n = 113) of them reported getting weight related advice. This is a global problem. A trend analysis of behavioral risk factors in the United States and a recent study on overweight and obese elderly in Unites States have shown identical proportions of individuals not being advised about weight by health professionals [32,33]. Moreover, majority of overweight/obese participants with comorbid conditions were not given weight advice by health professionals (n = 56, 78%) and most of the participants who were not advised misperceived their weight (n = 45, 80%). Physician's advice is a strong motivator to weight loss attempts as shown in multiple previous studies [32,34], therefore it is assumed that it would lead to improved perception of weight. However, it did not improve perception in our study. Probable reasons may be the powerful role of social demographic and personal factors like body size satisfaction in weight perception.

Low socioeconomic status is associated with weight misperception in many populations [8,11]. In our study, education was taken as proxy marker for socioeconomic status and we did not find any association between weight misperception and level of education.

Body size dissatisfaction is another important determinant of disordered weight control practices besides misperception of weight. Anderson et al., observed that women who were not satisfied with their body size were approximately nine times and women who were satisfied with their body size were three times more likely to try to lose weight as compared with women who were very satisfied with their body size [20]. It has been observed that greater body size satisfaction is associated with healthy lifestyle behavior and less weight gain in later years in children and adolescents [35]. Besides distorted perception, body dissatisfaction is strongly associated with increasing BMI and can lead to inappropriate weight control practices like binge eating and anorexia [36].

As the epidemic of obesity becomes global, it is imperative that steps are taken to control it. Obesity management now covers a wide range of long term strategies ranging from prevention, through weight maintenance and management of obesity comorbids to weight loss [4]. Therefore it is important for health professionals to identify overweight/obese people, educate them about the health risks of obesity and advise them about appropriate strategies for weight loss. More importantly concerted efforts need to be undertaken to prevent weight gain by emphasizing healthy eating habits and adequate exercise in children [37], adolescents and adults [4].

Our study has limitations. The study took place in an urban setting and hence these results cannot be generalized to the entire Pakistani population. But it can be assumed that the misperception rates may be higher than the rates observed in our study. There were more male participants than females probably because we included patients as well as attendants and female patients are usually accompanied by male attendants in our setting. We did not study body size dissatisfaction, which is another important determinant of weight loss. Instead, we enquired about their feelings regarding weight which we used as an indirect measure of body size dissatisfaction.

## Conclusion

We found that in this cross sectional survey, there was marked discordance between the actual and perceived weight. This was prevalent in older people, men, people who were happy with their weight and people who did not know their ideal body weight. Accurate perception is critical for individuals to be receptive to messages regarding weight maintenance or weight loss goals. Therefore, there is a need to educate people about healthy weights and correct weight perception as well as prevention of weight gain are important steps towards addressing the issue of obesity in Pakistan.

## Competing interests

The authors declare that they have no conflict of interest.

## Authors' contributions

SB conceived the idea, designed the study, analyzed the data and drafted the manuscript. FS performed the literature search, collected, cleaned, entered and validated the data and assisted in manuscript writing. AKK critically reviewed the data analysis and interpretation, contributed to revisions of the manuscript and provided conceptual feedback throughout. IA supervised the data analysis and critically reviewed the manuscript. KK further developed the initial idea and provided critical feedback in study design and manuscript. All authors read and approved the final version of the manuscript.

## Pre-publication history

The pre-publication history for this paper can be accessed here:

http://www.biomedcentral.com/1471-2458/11/363/prepub
